# Cold-priming of chloroplast ROS signalling is developmentally regulated and is locally controlled at the thylakoid membrane

**DOI:** 10.1038/s41598-019-39838-3

**Published:** 2019-02-28

**Authors:** Jörn van Buer, Andreas Prescher, Margarete Baier

**Affiliations:** Plant Physiology, Freie Universität Berlin, Dahlem Centre of Plant Sciences, Königin-Luise-Straße 12-16, 14195 Berlin, Germany

## Abstract

24 h exposure to 4 °C primes *Arabidopsis thaliana* in the pre-bolting rosette stage for several days against full cold activation of the ROS responsive genes *ZAT10* and *BAP1* and causes stronger cold-induction of pleiotropically stress-regulated genes. Transient over-expression of thylakoid ascorbate peroxidase (*tAPX*) at 20 °C mimicked and *tAPX* transcript silencing antagonized cold-priming of *ZAT10* expression. The *tAPX* effect could not be replaced by over-expression of stromal ascorbate peroxidase (*sAPX*) demonstrating that priming is specific to regulation of tAPX availability and, consequently, regulated locally at the thylakoid membrane. Arabidopsis acquired cold primability in the early rosette stage between 2 and 4 weeks. During further rosette development, primability was widely maintained in the oldest leaves. Later formed and later maturing leaves were not primable demonstrating that priming is stronger regulated with plant age than with leaf age. In 4-week-old plants, which were strongest primable, the memory was fully erasable and lost seven days after priming. In summary, we conclude that cold-priming of chloroplast-to-nucleus ROS signalling by transient post-stress induction of *tAPX* transcription is a strategy to modify cell signalling for some time without affecting the alertness for activation of cold acclimation responses.

## Introduction

Most plants of the temperate climate zones are adapted to annual and diurnal temperature variations. They can acclimate to slowly decreasing temperatures and to persisting cold^1–3^, but are harmed by sudden, short cold snaps^4,5^. Cold inhibits the Calvin-Benson-Cycle stronger than photosynthetic electron transport^6,7^. The imbalance between the two photosynthetic processes supports generation of reactive oxygen species (ROS) at the thylakoid membrane^6–8^. Additionally, cold decreases membrane fluidity, endangers membrane integrity and affects membrane protein function^9–11^. The impact of cold stress on plant growth and fitness can be severe. For example, three cold days in April 2017 (after a warm start into spring) destroyed up to 95% of the apple and cherry blossoms in Germany´s main fruit cultivation areas close to the Lake Constance, Hamburg (Altes Land) and Berlin (Havelland) and caused an average harvest loss of 46%^12^. Upon prolonged cold, acclimation processes re-establish photostasis, adjust membrane fluidity and accumulate protectants, such as osmolytes^9,13–16^. Regulation of metabolism and gene expression starts within minutes^17,18^, but it takes several days to establish full protection of the plants against chilling and freezing stress^4^. Induction and maintenance of acclimation are widely under control of the ICE1 (At3g26744)-CBF (C-repeat binding factor)-pathway^19^. It is costly to keep plants acclimated. Consequently, deacclimation starts as soon as the temperature increases and consumes cold-protective metabolites quickly^20^. For example, in *Arabidopsis thaliana* var. Col-0, about 90% of cold-induced carbohydrates are metabolized and gene expression is widely reset within 24 h at optimal growth temperatures^21,22^.

As shown recently, a single short cold period of 24 h at 4 °C primes *Arabidopsis thaliana* independent from activation of cold acclimation and modifies its response to future stresses^23,24^. In cold-primed plants, the pleiotropically stress regulated genes *CHS* (chalcone synthase; At5g13930) and *PAL1* (phenylalanine ammonium lyase; At2g37040) were stronger activated by a second cold stimulus that was applied 5 days after the 24 h priming cold stimulus. During the lag-phase between the two stresses, the transcript levels of *CHS* and *PAL1* were fully reset within the first 24 h at 18–20 °C. They were kept low, until the triggering cold stimulus reactivated their expression. Induction of the chloroplast ROS marker genes *ZAT10* (C2H2 zinc finger transcription factor; At1g27730) and *BAP1* (BON association protein 1; At3g61190) was almost entirely blocked in primed plants upon the second (=triggering) 24 h cold stress at 4 °C^24^. Such a modification of the response to a future stress depending on a previous stress over a stress-free period characterizes priming^25^.

Weakening of the priming effect in a *sAPX* (stromal ascorbate peroxidase; At4g08390) knock-out line of *Arabidopsis thaliana* and inversion of the effect in a *tAPX* (thylakoid ascorbate peroxidase; At1g77490) knock-out line pointed out a regulatory function of chloroplast ascorbate peroxidases in memorizing the priming stimulus^24^. The genes for *ZAT10* and *BAP1*, which are part of the plant environmental stress control system^24,26^, respond to chloroplast superoxide and singlet oxygen signals^27,28^. The signal transduction is still under investigation. As shown in stomata, the SAL1 (At5g63980)-PAP (3′-phosphoadenosine-5′-phosphate)-pathway mediates *ZAT10* induction in high-light^29^. Analysis in the genetic background of the protochlorophyllide accumulating *flu1* mutant showed that *BAP1* is under control of the EXECUTER pathway^30^. Besides ROS signalling, cold activates the HOS1 (At2g39810) and OST1 (At4g33950) controlled ICE1-CBF pathway and hormone and metabolic signalling, which cross-talk with ROS signalling^19,26^.

Priming phenomena have been described for the response to various biotic and abiotic stresses, including cold^23,25,31,32^. The examples have in common that the stress stimuli were too short or too weak to establish acclimation. Priming typically sets metabolic marks (elicitor factors) or chromatin modifications, which affect signal transduction and gene expression when the plants are triggered by a second stress stimulus^23,25,33^. Compared to acclimation, which binds large amounts of resources in protection (which could otherwise support growth), the metabolic costs of priming are assumed to be low^21,23,34^. But even priming can be costly, if it restricts the stress sensitivity or the reaction potentials^35,36^. Consequently, there is a necessity for “extinction” or at least for an option for “overwriting” of the stress memory required to re-establish stress responsiveness after some time and to avoid exhaustion by accumulative memory formation in response to multiple priming events^35,36^.

In our initial analysis of cold-priming in *Arabidopsis thaliana*^24^, we showed cold-priming of ROS-responsive genes in 4-week-old plants. The plants had formed several rosette leaves, but were still far from initiation of bolting under short-day conditions. If priming competes with growth for resources, the memory should be extinguished or at least weakened before bolting starts to avoid loss of reproductive fitness. In the present study, we analysed cold-priming and the priming stability in the seedling, the pre-bolting and the highly bolting activation-sensitive stage of 2-, 4- and 6-week-old Arabidopsis plants and in young, intermediate and old leaves of 6 week old plants. We show that the primability is regulated more by plant age than by leaf age and correlates with *tAPX* transcript abundance regulation in response to the priming cold stress. Priming analysis in inducible chloroplast *APX* over-expresser and silencing lines gave causal evidence that cold-priming is specifically regulated by post-cold regulation of *tAPX* expression.

## Results

### Developmental regulation of cold-priming

**Figure 1:**
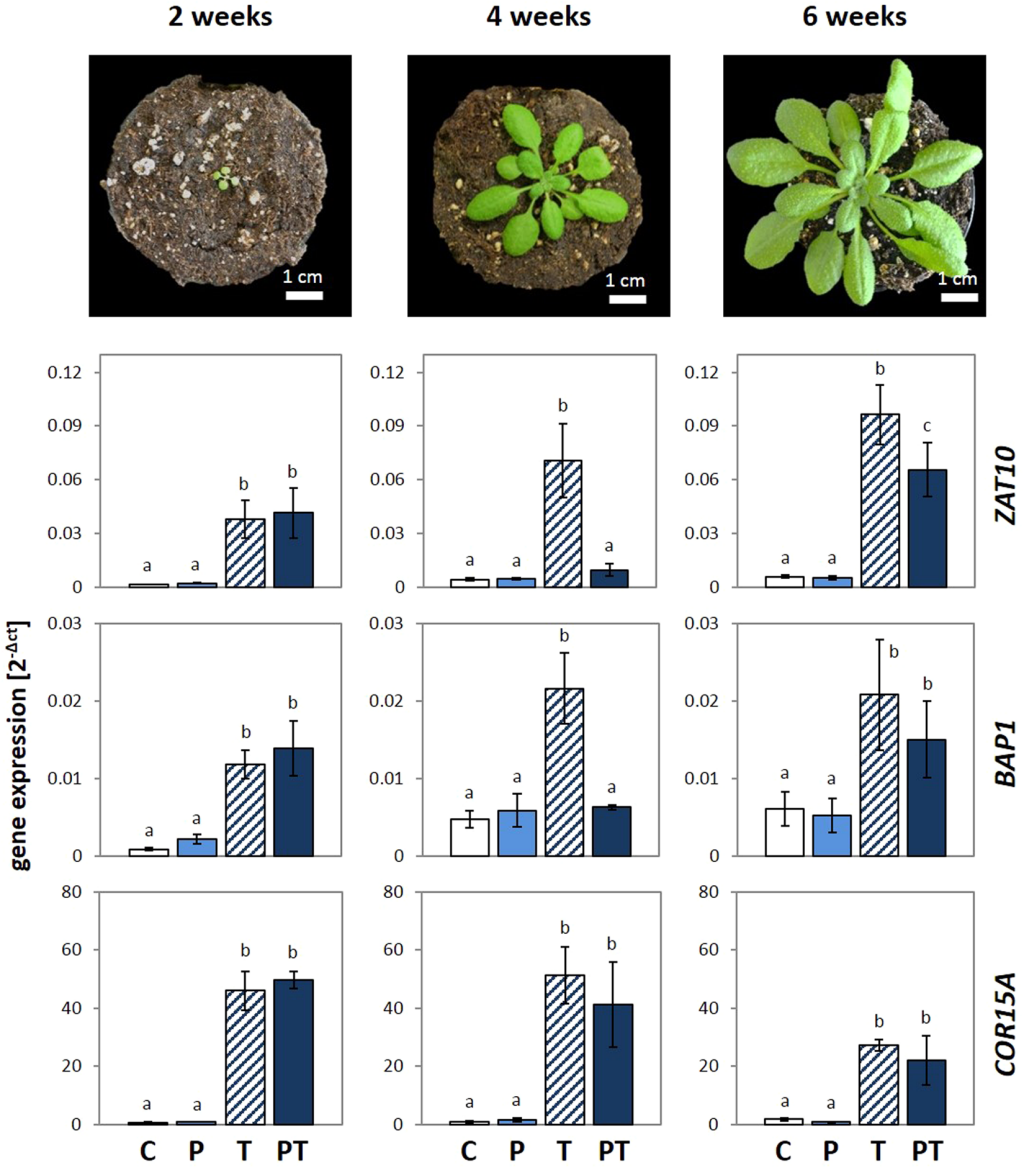
The effect of plant age on priming of *ZAT10* and *BAP1*. *Arabidopsis thaliana* var. Col-0 were primed at an age of 2, 4 and 6 weeks by a 24 h cold-treatment at 4 °C. The 24 h 4 °C triggering stress was applied 5 days after priming. The transcript abundance for the primable ROS marker genes *ZAT10* and *BAP1* was evaluated directly after triggering in primed and triggered (PT), only primed (P), only triggered (T) and in control plants (C) and normalized to the geometric mean of the transcript levels of two constitutively expressed genes. As control for monitoring the cold-responsiveness, the transcript levels of the non-primable cold marker gene *COR15A* were determined. The letters refer to distinct significance groups as determined by ANOVA (Tukey’s test, p < 0.05, n = 3 ± SD).

Cold-priming of ROS-signalling was previously analysed in 4-week-old Arabidopsis plants that are in the rosette stage with 12–15 leaves (stage 1.14^37^), of which more than 80% were still growing in length and width. To test if changes in chloroplast function and metabolism could affect the primability during leaf and plant development, we analysed 2-, 4- and 6-week-old Arabidopsis plants 5 days after cold-priming for priming effects on cold induction of *ZAT10* expression. The 2-week-old plants were in the transition from the cotyledon stage to the rosette stage (stage 1.02^37^) and had just formed the first pair of primary leaves (Fig. 1). At this stage, the seed resources are widely consumed and growth depends on carbohydrate biosynthesis^38–40^. The 6-week-old plants were in stage 3.70 to 3.90^37^ (Fig. 1). The oldest leaves had reached their maximum size, while new leaves were still formed in the centre of the rosette (Fig. 1).

Prior to analysis of priming effects, the relative cold inducibility of the marker genes was analysed in naïve plants at the time primed plants were triggered (T-plants; Fig. 1). *ZAT10* und *BAP1* were, like the ICE1-CBF-controlled gene *COR15A* (cold-regulated gene 15A; At2g42540)^41,42^, cold-inducible in all three tested developmental stages, but the induction intensity varied with plant age in a gene-specific manner. For *ZAT10*, the intensity of cold induction steadily increased with age (Fig. 1). The cold responsiveness of *BAP1* increased from the youngest to the medium old plants, but not further. The induction of *COR15A*, that encodes a chloroplast protein protecting the inner envelope membrane^43^ and served as a reference gene for the not-primable ICE1-CBF pathway^24^, was high in the youngest and in the medium old rosettes, but weak in the oldest plants.

In plants, which were primed for 24 h at 4 °C at an age of 2 weeks, neither *ZAT10*, representing the O_2_^−^/H_2_O_2_ signalling pathway^44^, nor *BAP1*, which responds to the singlet oxygen signals^44^, differed in their response between “triggered only” (T) and “primed and triggered” (PT) plants (Fig. 1). 6-weeks-old plants showed much weaker priming effects (PT/T = induction level in primed and triggered (PT) plants relative to only triggered ones (T)) on cold induction of *ZAT10* than 4-week-old plants (Fig. 1). *BAP1* did not show a priming effect in the 6-week-old plants. These regulation patterns demonstrated that cold-primability is established at an age between 2 and 4 weeks and fades out later during development. *COR15A*, which is transcriptionally induced by CBFs^45,46^, was not primable at any age.

### Age-dependent priming regulation within the rosette of 6-week-old plants

**Figure 2:**
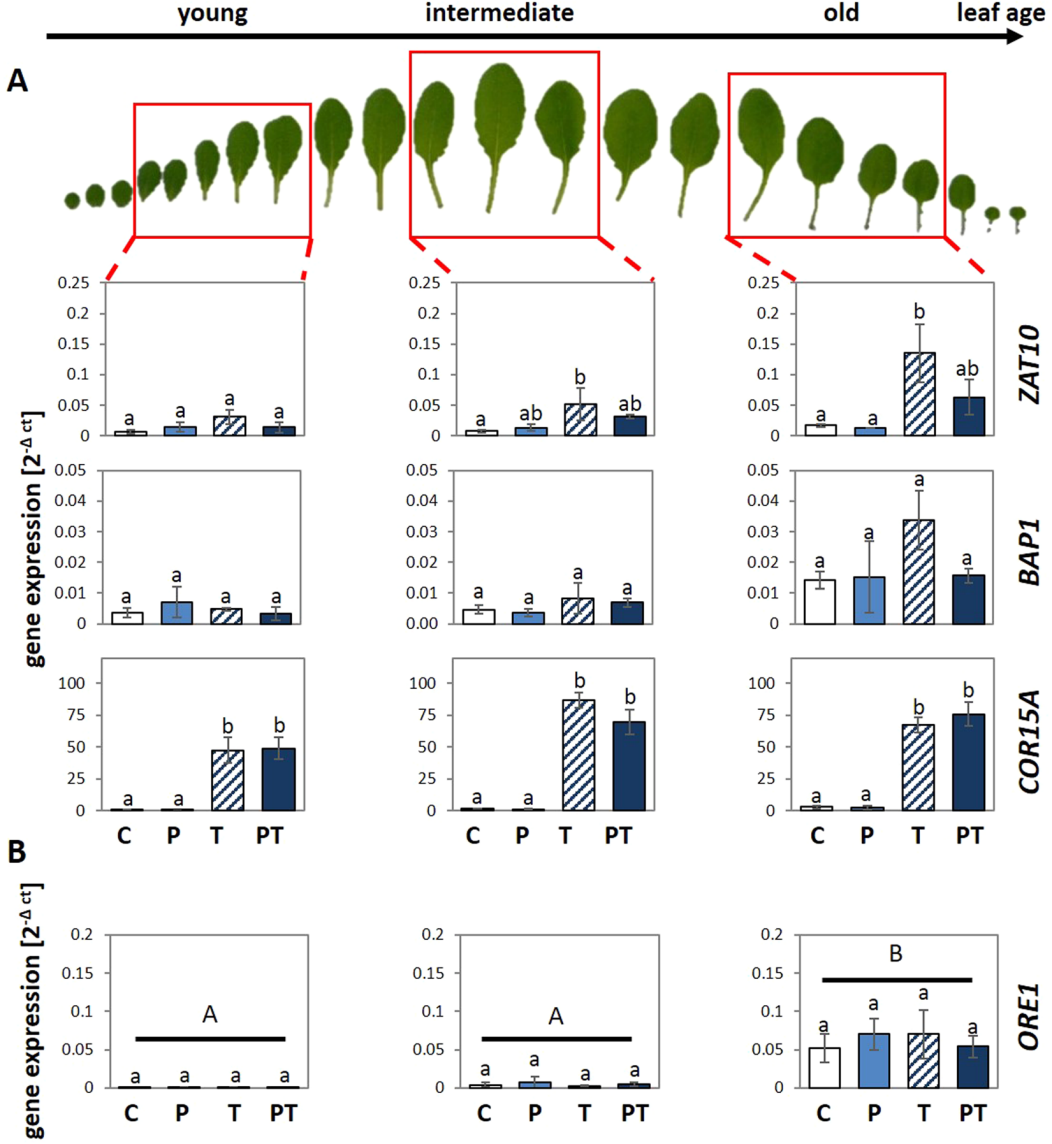
Priming of leaves in different developmental stages of a 6-week-old rosette. Six week old plants were primed and triggered according to the experimental design (Fig. 7) and harvested after the triggering stimulus was applied. (**A**) The transcript abundance was measured for the primable ROS marker genes *ZAT10* and *BAP1* and normalized to the geometric mean of two constitutively expressed genes. Additionally the non-primable cold marker gene *COR15A* was analysed. (**B**) Transcript levels of the early senescence gene *ORE1* were determined in the same samples as quantitative measure for the onset of senescence. An ANOVA (Tukey’s test, p < 0.05, n = 3 ± SD) was performed. The small letters refer to significance groups with leaf sets of the same age and different capital letters show significant differences between different age groups.

To analyse if the weaker primability in 6-week-old plants is linked to specific leaves or characteristic for the entire rosette, the priming effects on *ZAT10* and *BAP1* transcript levels were compared in young, intermediate and old leaves of 6-week-old Arabidopsis plants (Fig. 2). The oldest leaves expanded between the 2nd and 3rd week of growth (in stage 1.02–1.03) and formed the main leaf biomass in 4-week-old plants. The medium old ones formed the centre of the rosette of 4-week-old plants and were at this stage of development less than 8 mm long, similar to the youngest leaves of 6-week-old-plants. Although the *ZAT10* and *BAP1* transcript levels did not differ significantly between PT and T plants in the three leaf sets, they showed in all biological replicates a trend towards stronger primability in the oldest leaves of the 6-week-old-plants (Fig. 2A). *COR15A* was strongly cold-inducible in all leaves, but not sensitive to 24 h cold-priming in any of the three leaf groups (Fig. 2A).

### Regulation of onset of senescence and sugar distribution

**Figure 3:**
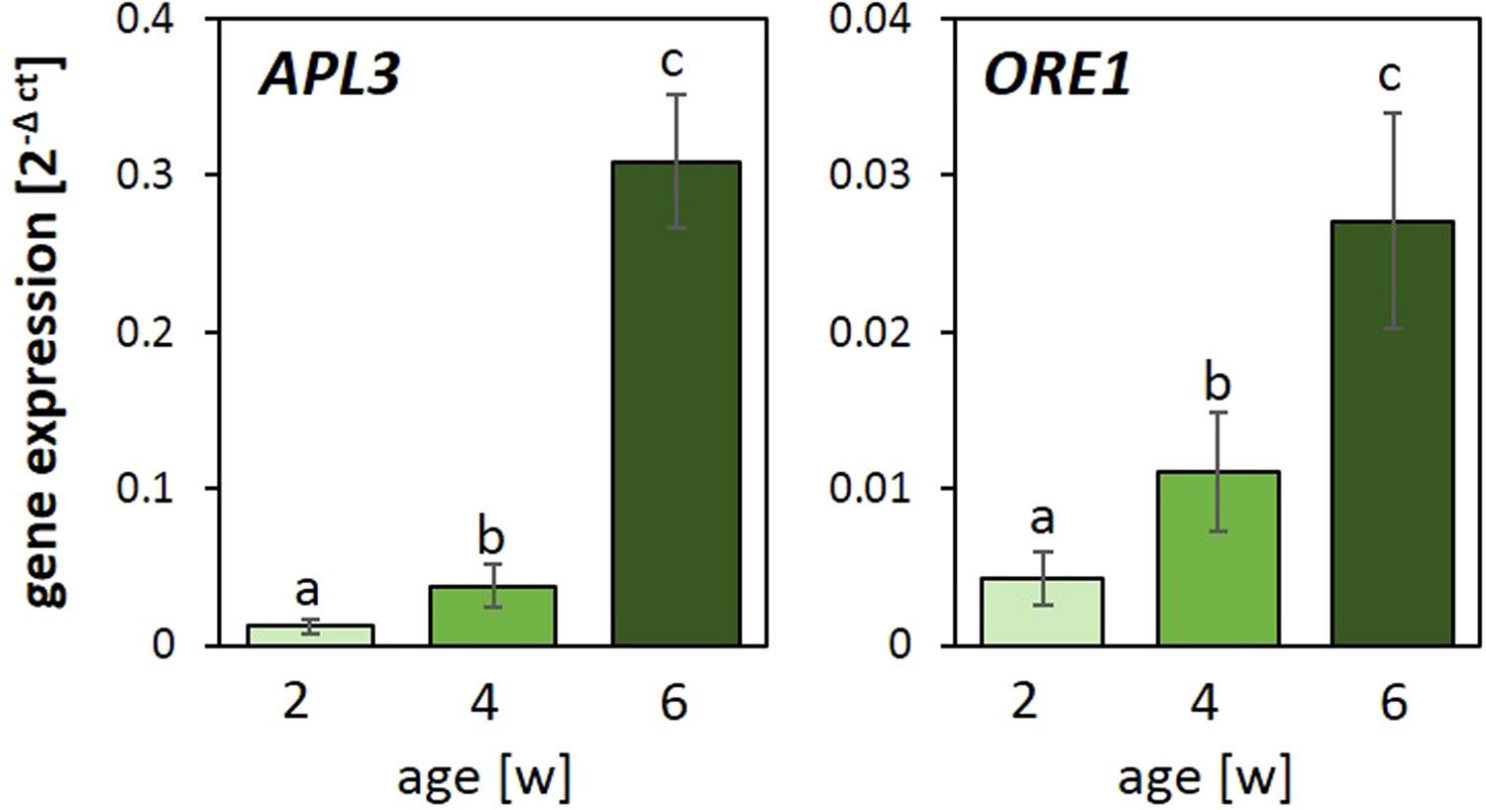
Normalized transcript abundance of *APL3* and *ORE1* in 2-, 4- and 6-week-old rosettes. The transcript abundance of the carbohydrate sensitive gene *APL3* and senescence marker gene *ORE1* were determined in 2-, 4- and 6-week-old rosettes and normalized to the transcript levels of two constitutively expressed genes. The letters refer to distinct significance groups as determined by ANOVA (Tukey’s test, p < 0.05, n = 3 ± SD).

The transcript levels of *APL3* and *ORE1* were analysed as markers for the physiological status of the plant material (Fig. 3). *APL3* (At4g39210) encodes the large subunit of ADP-glucose pyrophosphorylase and supports synthesis of transitory starch in chloroplasts in the feast status^47,48^. Its expression is induced in the leaf blade upon excess carbohydrate availability and characterizes the carbohydrate storage status of leaves^48,49^. In our study, *APL3* expression was low in 2-week-old plants, slightly higher in 4-week-old ones and strongly elevated in 6-week-old plants (Fig. 3 left) demonstrating that 2-week-old plants were still in the sink-status, 4-week-olds were just in the process of accumulating excess starch and 6-week-olds had a strong carbohydrate storage setting.

Activation of *ORESARA 1* (*ORE1*, *ANAC092*; At5g39610) marks the onset of senescence prior to the occurrence of visible phenotypes, like chlorosis^50–52^. The transition process involves an increase in the sensitivity to chloroplast ROS^53^ and chlorophyll degradation^54^. In the youngest plants, *ORE1* transcript levels were almost not detectable (Fig. 3 right). Consistent with the work by Kim and co-workers^55^, *ORE1* transcripts started to accumulate in 4-week-old plants (Fig. 3). Within the next two weeks, the transcript level more than doubled demonstrating manifestation of the transition.

*ORE1* expression was very low in the youngest leaves of 6-week-old plants, only weakly expressed in the intermediate old leaves and activated in the oldest leaves of 6-week-old plants (Fig. 2B). The transcript abundance patterns of *APL3* and *ORE1* relative to leaf age resembled that of 2-, 4- and 6-week-old plants, demonstrating comparability of the two experimental set-ups of our study (Figs 1 and 2) with respect to carbohydrate and senescence regulation.

*ORE1* transcript abundance regulation was not cold-sensitive. Comparison of the transcript levels in T- and PT-plants gave also no indication that the gene is cold-primable (Fig. 2B). The similarity of the *ORE1* transcript levels in C, P, T and PT-plants (Fig. 2B) demonstrated that the 24 h 4 °C priming stimulus did not induce or accelerate aging.

### Specificity of tAPX regulation in cold-priming of ROS signalling

**Figure 4:**
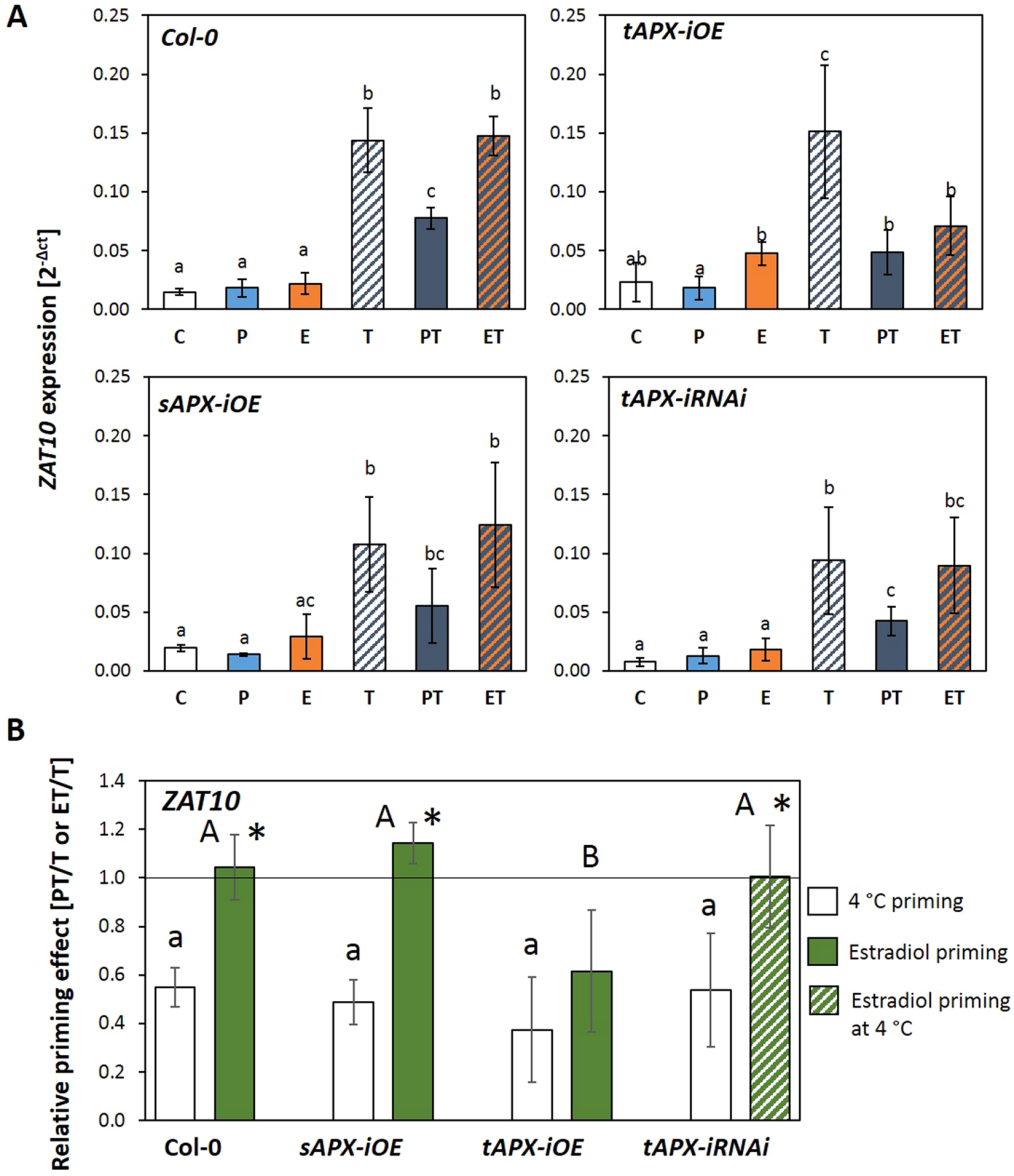
The effect of cold or deregulation of plastidic ascorbate peroxidases on a subsequent cold trigger. (**A**) *ZAT10* transcript levels in control plants (**C**), only cold-primed (P), only estradiol treated (**E**), only cold triggered (T) and cold-primed and cold-triggered (PT) and estradiol-treated and cold-triggered (ET) *Col-0*, *sAPX-iOE*, *tAPX-iOE* and *tAPX-iRNAi* plants of the same age. The *tAPX*-iRNAi ET plants were cold primed and sprayed with estradiol. The letters refer to distinct significance groups as determined by ANOVA (Tukey’s test, p < 0.05, n = 4 ± SD). (**B**) Priming effect. *ZAT10* transcript abundance in cold (white) or by estradiol spraying (green) primed Col-0, sAPX-iOE and *tAPX*-iOE and *tAPX*-iRNAi lines after 24 h cold triggering (PT and ET, respectively) normalized on the transcript abundance in triggered only plants (T-plants). The *tAPX*-iRNAi plants were cold-primed and sprayed with estradiol (green-white striped). The crude data are identical to those in section A. for calculation of the means, standard deviations and the statistical analysis (one-sided t-Test p < 0.05; n = 4) the PT/T- and ET/T-ratios, respectively, were calculated independently for each biological replicate first. Different small letters show significance of difference in cold primability, different capital letters difference in the cold response after estradiol spraying. The asterisks label significantly different results between cold- and estradiol-priming.

In cold-primed plants, *tAPX* transcripts and proteins accumulated in the post-stress phase^24^. The priming effect of 24 h of cold (4 °C) on *ZAT10* and *BAP1* expression was inverted in *tAPX* knock-out lines and weakened in *sAPX* knock-out lines pointing out that *tAPX* is of stronger importance for setting and maintenance of the priming memory than *sAPX*^24^. The catalytic subunits of the two chloroplast APX isoforms are highly conserved^56^ As shown in *sAPX* and *tAPX*-knockout lines, the two enzymes can compensate for the loss of the respective other one under low stress conditions^57–61^. To differentiate the functions of the closely related genes for tAPX and sAPX in mediating priming, we tested whether the priming effect of a 24 h 4 °C cold pre-treatment can be mimicked in absence of cold solely by transient *tAPX* over-expression or also by *sAPX* over-expression (Fig. 4). We induced expression of *tAPX* and *sAPX* full-length constructs at 20 °C in 4-week-old *Arabidopsis thaliana* using an estradiol-inducible system^62^. As an inverse approach, cold-induced accumulation of *tAPX* transcript levels was antagonized by transient silencing of *tAPX* expression in an estradiol-responsive *tAPX*-RNAi (RNA interference) line^60^ after a 24 h 4 °C cold stimulus. *tAPX* and *sAPX* transcript levels were monitored by qPCR prior to application of the cold trigger (Suppl. 1 and 2) and *ZAT10* transcript abundances were analysed before and after triggering (Fig. 4).

All *APX* transgenes were strongly inducible by estradiol (Suppl. 1 and 2). Western-Blots demonstrated that induction of the *sAPX-* and *tAPX*-iOE (induced overexpresser) constructs increased the APX protein levels (Suppl. 1). The apparent molecular size of the sAPX and tAPX proteins corresponded to that of the mature chloroplast forms reflecting processing of the import signal and, consequently, translocation of the proteins into chloroplasts (Suppl. 1). The estradiol-treatment itself did not affect cold-induction of *ZAT10* in Col-0 plants (Fig. 4A; comparison of only cold-treated T- and estradiol- and cold-treated ET-plants). In all three transgenic lines, the gene constructs were (slightly) active in absence of estradiol (Suppl. 1 and 2). The construct leakiness did not affect *ZAT10* transcript levels under control conditions in any of the lines (Fig. 4A; C-plants), but the *ZAT10* transcript levels were slightly (but not significantly) increased in estradiol-treated plants (Fig. 4A; E-plants). The *ZAT10* transcript levels were decreased in estradiol-treated *tAPX-iOE* plants to similar levels as in cold-primed plants of the same line. Confirming the regulatory function of *tAPX* expression in mediating priming, the *ZAT10* transcript levels were not decreased in the *tAPX-iRNAi* line after cold pretretament and cold triggering (Fig. 4A; comparison of ET- and PT-plants). Normalization of the PT- and ET-values on the T-value in each independently cultivated and treated biological replicate and calculation of the means and standard derivation between the biological replicates eliminates part of the unspecific background variation (Fig. 4B). The normalized data confirmed that the cold-priming effect on *ZAT10* regulation can be mimicked by transient induction of *tAPX* (*tAPX-iOE*-line) and blocked by transient silencing of *tAPX* (*tAPX-iRNAi*-line). Estradiol-induced *sAPX* overexpression, which is the stromal counterpart of tAPX with a conserved catalytic domain^56^, had no effect on the cold response of *ZAT10*.

### Regulation of *tAPX* promoter activity

**Figure 5:**
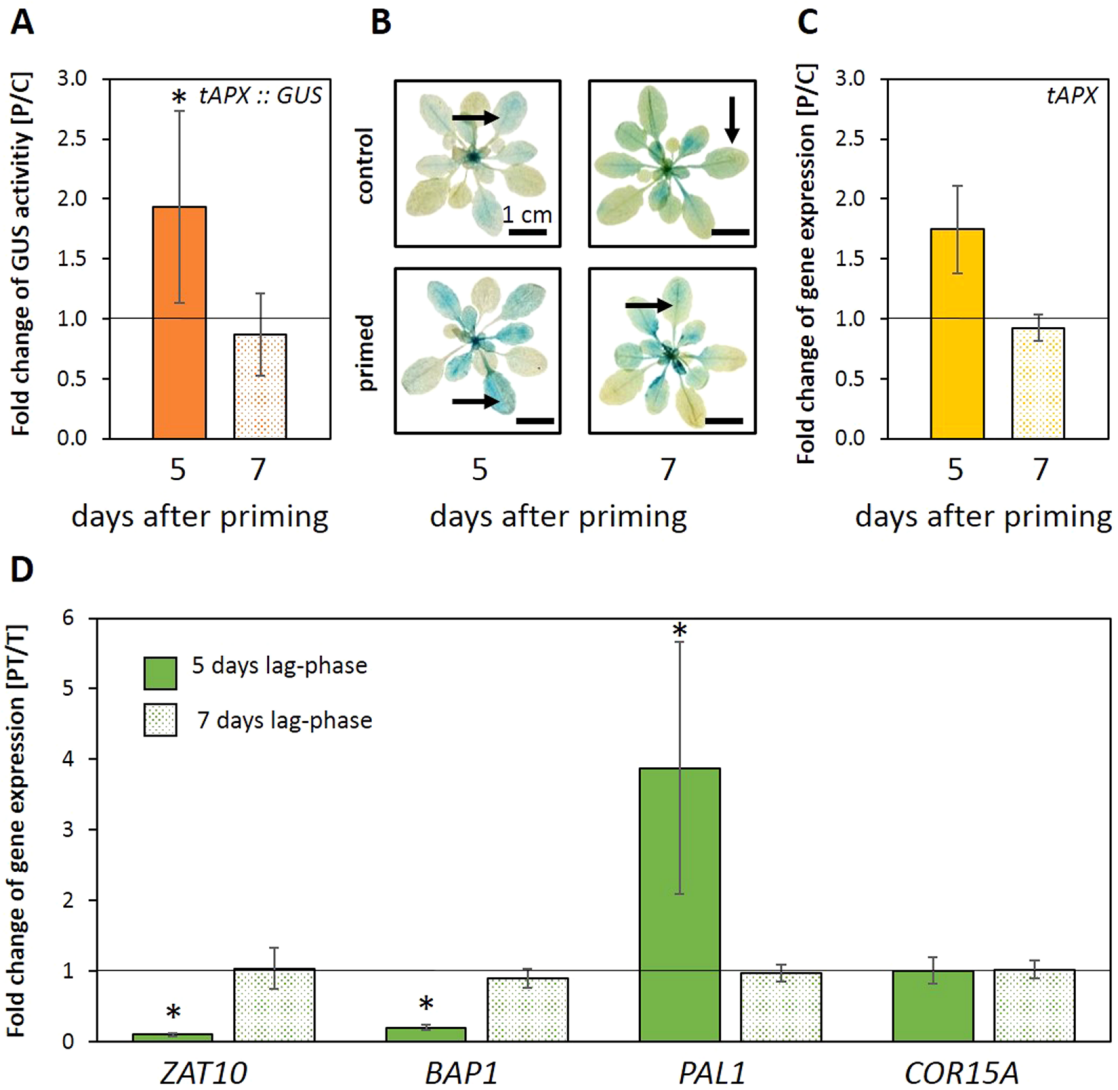
The effect of a prolonged lag-phase of 7 days on primable genes and *tAPX* expression. (**A**) Quantification of GUS activity in 4-week-old rosettes 5 (orange bar) or 7 (dotted bar) days after priming, respectively. The graph depicts the specific activity in primed plants at the end of the lag-phase relative to the specific activity in control plants (n = 10; mean ± SD, * t-Test p < 0.05. (**B**) Representative GUS staining pattern of *tAPX*_prom_::*GUS* plants (n = 10) 5 or 7 days after cold-priming and in control plants. The arrows marks the leaf stage that was used for the quantification of GUS activity. (**C**) Comparison of the normalized *tAPX* transcript abundance 5 and 7 days after priming relative to the transcript levels in control plants (n = 3; mean ± SD, * one-sided t-Test p < 0.05). (**D**) Normalized transcript levels of *ZAT10*, *BAP1*, *PAL1* and *COR15A* in PT-plants relative to T-plants at the time-point directly after the end of the triggering stimulus after a lag-phase length of either 5 days (green bar) or 7 days (dotted bar) (n = 3; mean ± SD, * one-sided t-Test p < 0.05).

The catalytic site of tAPX, like that of other chloroplast antioxidant enzymes, e.g. sAPX and peroxiredoxins, is sensitive to inactivation upon stressful conditions^63,64^. Consequently, *de novo* tAPX synthesis is required to maintain the enzyme activity level in chloroplasts^65^. To test if stronger transcription of the nuclear located *tAPX* gene is involved in the priming response we performed full factorial priming assays with C, P, T and PT-plants^24^ in 4-week-old plants of a reporter gene line expressing a fusion protein of GFP (green-fluorescent protein) and GUS (glucuronidase) under the control of the *tAPX* promoter (*tAPX*_prom_::*GFP*-*GUS*). 5 days after priming, higher GUS activities were observed in cold primed plants (Fig. 5A). After priming, the *tAPX* promoter was strongest activated in the medium old leaves by priming (arrow in Fig. 5B), although the background transcription activity was highest in the youngest leaves.

### Stability of the priming memory in wildtype plants

7 days after priming, *tAPX* transcript levels and *tAPX* promoter activity were (almost) back to the levels detected in naïve plants of the same age (Fig. 5A,C). Consistent with the decrease in *tAPX* promoter activity, the priming effects were lost for *ZAT10* and *BAP1* (Fig. 5D). In the original publication on cold-priming of genes in 4-week-old Arabidopsis plants^24^, we also reported stronger cold activation of pleiotropically stress regulated genes, such as *PAL1*, 5 days after the priming treatment. Like *ZAT10* priming, the expression promoting priming effect on *PAL1* was lost 7 days after the cold pre-treatment (Fig. 5D). *COR15A* expression showed no priming response after a lag-phase of 7 days, as after a lag-phase of 5 days (Fig. 5D).

**Figure 6:**
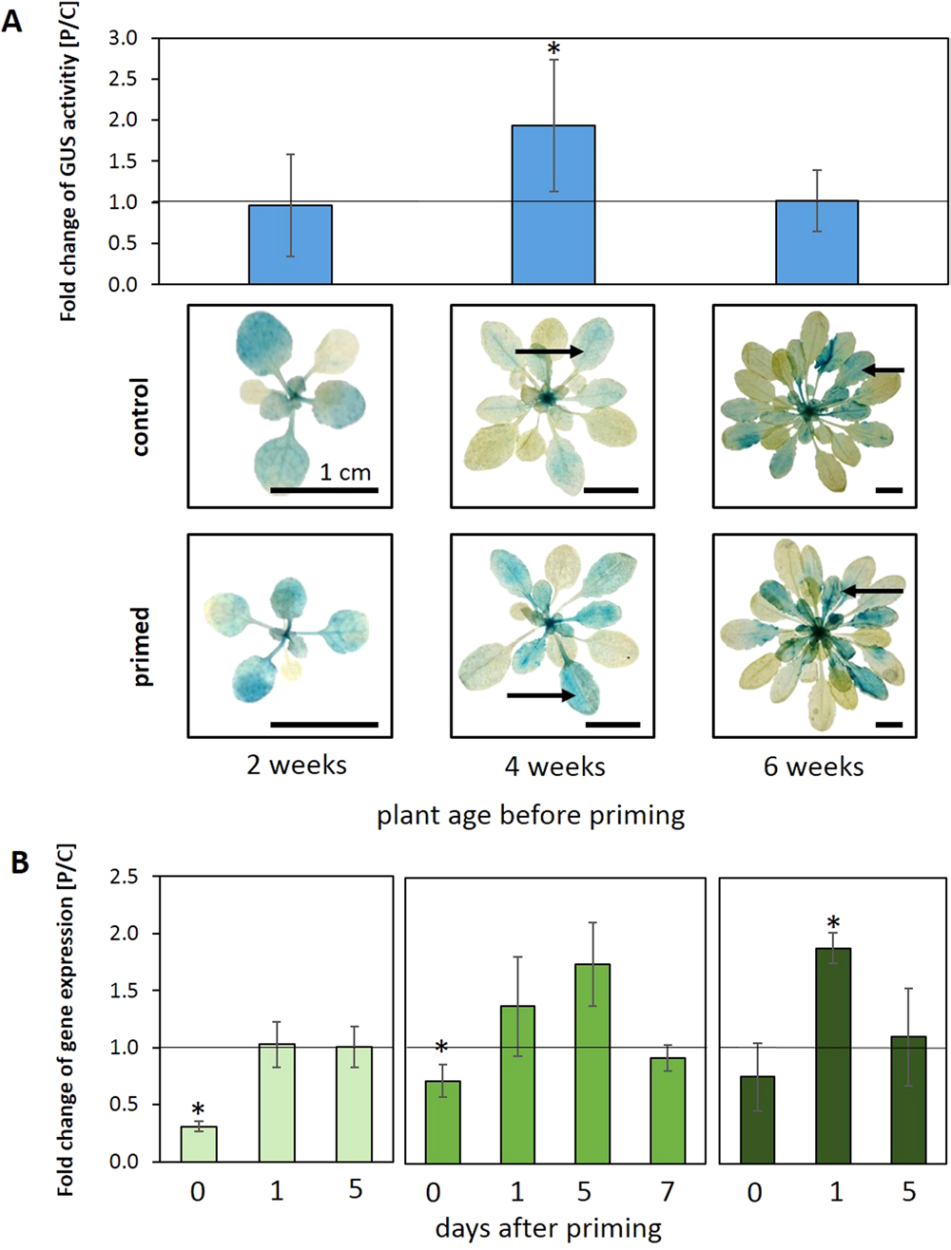
*tAPX* regulation in response to the priming stimulus. (**A**) The GUS activity in 2-, 4- and 6-week-old rosettes of primed *tAPX*_prom_::*GUS* reporter gene plants 5 days after priming relative to the activity in untreated control plants of the same line (n = 10; mean ± SD, * t-Test p < 0.05). (**B**) GUS staining patterns of representative plants out of 10 individuals 5 days after priming (bottom) and un-treated controls (top) at different ages (2-, 4- and 6-week-old). The arrow indicates the developmental stage of leaves used for the GUS activity measurements. (**B**) *tAPx* transcript levels 0, 1 and 5 days (and 7 days additionally for 4-week-old plants) after priming relative to the levels of parallel cultivated untreated plants (n = 3; mean ± SD, * one-sided t-Test p < 0.05).

The analysis of *tAPX* expression and memory stability regulation was extended to 2- and 6-week-old plants (Fig. 6). In these younger and older plants, *tAPX* promoter activity (analysed as GUS activity) was not increased 5 days after cold-priming (Fig. 6A). In 2-week-old plants, comparison of *tAPX* transcript level regulation (Fig. 6B) demonstrated that the *tAPX* transcript level decreased during 24 h at 4 °C to less than half of the level of naïve plants. Within the next 24 h, the transcript levels in cold-treated plants (P-plants) were indistinguishable from that in control plants (C-Plants). In 6-week-old plants, the *tAPX* transcripts accumulated on the first day of the post-stress phase to even higher levels than in 4-week-old plants, but declined to levels similar to that in naïve plants within 5 days. The comparison demonstrated, consistent with the GUS-staining patterns (reporting *tAPX* promoter activity) that 2-week-old plants did not activate *tAPX* expression after 24 h exposure to 4 °C to levels higher than prior to the priming cold stimulus and that 6-week-old plants lost the *tAPX* induction effect faster.

## Discussion

Throughout development, cold-priming of *ZAT10* regulation correlated with post-cold induction of *tAPX* expression in Arabidopsis wildtype plants (Figs 5 and 6). Furthermore, overexpression of *tAPX* at 20 °C mimicked and *tAPX* silencing (after a 4 °C treatment) antagonized cold-priming of *ZAT10* expression regulation (Fig. 4). The *tAPX* effect could not be replaced by stronger *sAPX* expression (Fig. 4), although the catalytic domains of sAPX and tAPX are highly conserved^56^. From these observations, we conclude that cold-priming is causally and specifically regulated by APX availability at the thylakoid membrane. Due to the large stromal domain, tAPX is located in the unstacked areas of thylakoids, where also photosystem I (PS-I) is placed^66,67^. From various studies, tAPX is known to be the main enzyme detoxifying H_2_O_2_ generated at the stromal site of PS-I^67–70^. However, it´s catalytic centre is sensitive to inactivation by ROS^71,72^. Recovery takes place by *de-novo* synthesis and depends on chloroplast-to-nucleus signalling, cytosolic translation and protein import into chloroplasts^56^. Additionally, tAPX accumulation decreases *ZAT10* induction upon stress, but does not antagonize *ZAT10* expression *per se*, as the comparison of C- and E-plants of the *tAPX-iOE* line at 20 °C and the comparison of C-plants of Col-0 and *tAPX-iOE* demonstrated (Fig. 4A). If tAPX availably is insufficient upon stress, H_2_O_2_ can escape from the thylakoid membrane^60,61^, accumulate in the stroma, diffuse into the cytosol and, finally, trigger extra-plastidic signalling cascades^73,74^. The primable genes *ZAT10* and *BAP1* sensitively respond to chloroplast ROS and are key regulators of plant stress signalling pathways^24,28,74,75^. They control vitally important stress responses like effector triggered immunity and induction of high light protection^26,74,76,77^. Attenuating *ZAT10* induction by priming specifies ROS-signalling and enables stronger cold induction of pleiotropically stress regulated genes, such as *CHS* and *PAL1*, after cold-priming^23,24,78,79^.

In our study, the cold-priming effect on *ZAT10* expression was independent of the metabolite status or senescence regulation, but regulated during rosette developmental (Figs 1–6). We think that the strong primability in the pre-bolting stage evolved in the context of the natural life cycle pattern. *Arabidopsis thaliana var*. Col-0 typically follows a winter annual growth regime^80^. Temperatures above 15 °C can shift the life history towards a rapid cycling one^80–82^. Consitently, the shoot apical meristem of *Arabidopsis thaliana* forms more than 40 leaves under short day conditions under optimal, stress free lab conditions^83,84^. Long-day conditions promote bolting. However, the shoot apical meristem is arrested in the vegetative stage even under bolting-promoting long-day conditions up to around 4 weeks^37,85^. In non-vernalized plants, the lengths of the juvenile and transition phase are genetically fixed^86^. Prior to bolting, Arabidopsis rather invests in growth and protection of already existing leaves than in formation of new leaves^37,87,88^ to support habitat occupation and to acquire resources and stability for inflorescence and fruit formation^89,90^. Arabidopsis bolts in spring after a series of unpredictable cold snaps. In the diversity of vitality promoting mechanisms, priming is assumed to be the least cost intensive one^23,25^. As shown for *COR15A*, it does not affect cold induction of canonically cold-regulated cold accumulation processes^1^ (Figs 1–3), but adjusts cell signalling in a very specific, developmentally controlled and temporally restricted manner.

## Conclusion

Cold-priming of chloroplast-to-nucleus ROS signalling is mediated by transcriptional regulation of tAPX availability in a developmentally controlled and erasable manner (Figs 1–6). We have postulated that cold-priming evolved as a specific strategy to manage stress signalling, when stresses occur in an unpredictable pattern and are too short to activate acclimation^24^. The catalytic site of the main regulator of the cold-priming memory, tAPX, is highly sensitive to inactivation by H_2_O_2_/ROS^63,64^. The “instability” of chloroplast APX against ROS characterizes *tAPX* as an ideal target for priming regulation: Firstly, the priming setting can be quickly erased upon severe stress by inactivation of tAPX, which avoids fixation into an inappropriate setting^35,36^. Secondly, the lability of tAPX makes priming depended on *de-novo* synthesis of tAPX. Transcription in the nucleus, translation in the cytosol, protein import into chloroplasts and embedding of tAPX into the thylakoid membrane enable fine-tuning and cross-talk with other signalling processes^38,39,65,91–93^. Thirdly, tAPX controls an electron dissipation pathway subordinated to thioredoxin and NADP^+^ reduction^94,95^. The tAPX-dependent water-water-cycle is of minor importance at low stress levels^59^, but has a key function in relaxing the electron pressure in the photosynthetic electron transport chain upon severe imbalances from photostasis^96^. In addition to chloroplast and cellular ROS levels, tAPX activity also controls electron flux into cyclic photosynthetic electron transport, non-photochemical quenching and plastoquinone reduction^28,57,97–99^. In our opinion, tAPX is a predetermined breaking point in the centre of the plant stress signalling network. The lability of the catalytic site of tAPX enables plants to switch upon prolonged cold periods from attenuating chloroplast ROS signalling and activating pleotropic stress responses to activation of canonical cold acclimation^1^. With the onset of acclimation, down-regulation of *tAPX* expression intensity^24,100^ can manifest the switch.

The ability to modify the sensitivity of selective stress signalling cascades^24^ in the trade-off between cold-acclimation, pleiotropic stress protection and growth after short or weak stresses, without blocking the responsiveness of signalling cascades mediating acclimation responses upon prolonged stress (Figs 1 and 2), characterizes priming and explains, in our opinion, manifestation of the process during evolution.

## Methods

### Plant material and growth conditions

**Figure 7:**
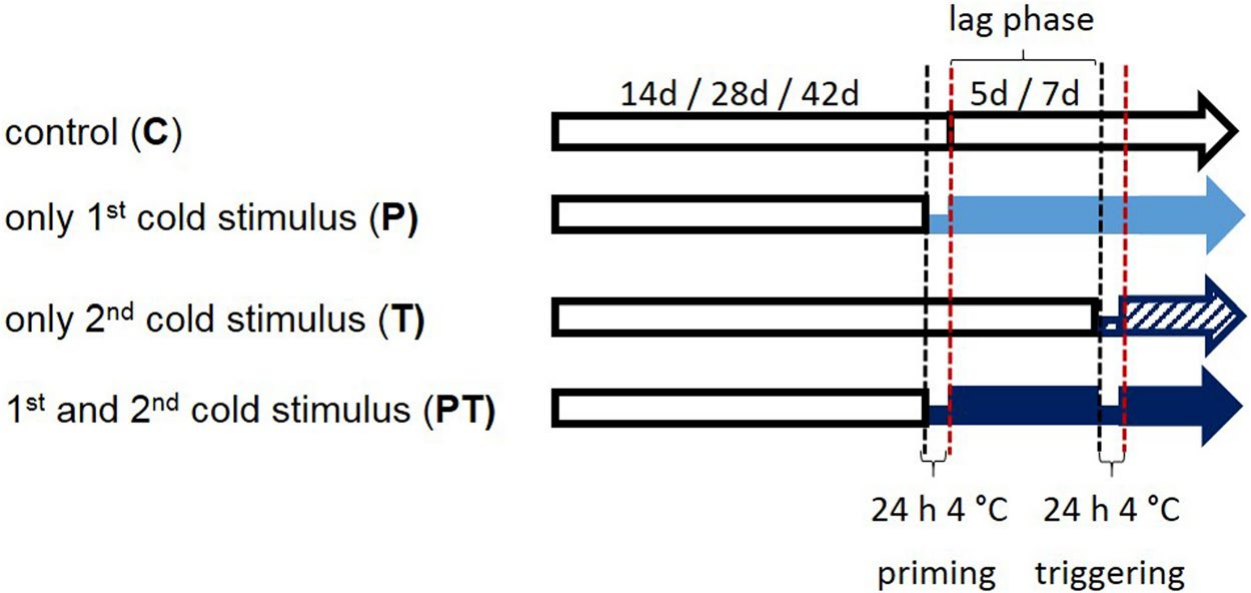
Outline of the priming experiments. Plants were either grown for 2, 4 or 6 weeks under control conditions, before half of the plants were cold-treated for 24 h at 4 °C (primed, P). Afterwards, the plants were transferred back to the standard growth conditions. Five or seven days later (lag-phase) half of the plants of each group was treated for 24 h at 4 °C (trigger, T). Twice cold-treated plants are referred to as “primed and triggered” (PT), once treated as “only primed” (only the earlier cold treatment) (P) or “only triggered” (only the later cold treatment) (T) and not cold-treated plants as controls (C).

*Arabidopsis thaliana* var. *Col-0* wildtype plants and transgenic lines were grown on Arabidopsis soil [70 volumes “Topferde” (Einheitserde, Sinntal-Altengronau, Germany), 70 volumes “Pikiererde” (Einheitserde, Sinntal-Altengronau, Germany), 25 volumes Perligran Classic (Knauf, Germany)] supplied with 0.5 g l^−1^ dolomite lime (Deutsche Raiffeisen-Warenzentrale, Germany) and 0,5 g l^−1^ Axoris Insekten-frei (COMPO, Münster, Germany). After stratification at 4 °C, the plants were cultivated for 2, 4 and 6 weeks in a growth chamber at 10 h light (20 °C, 95–110 μmol photons*m^−2^*s^−1^)/14 h dark (18 °C) cycles and a humidity of 60% ± 5% prior to priming (Fig. 7). All cold treatments were performed at 4 °C at the same light intensity and with the same fluorescent stripes (L36W/840 Lumilux Cool White; Osram, Munich, Germany), like in the previous study^24^. Biological replicates were cultivated and cold-treated independently.

The priming treatments were started 2.5 h after the onset of light and terminated exactly 24 h later to avoid circadian effects. After priming, the plants were transferred back into the 20 °C/18 °C growth regime. Plant material was harvested in primed and parallel grown control plants immediately after priming or 1, 5 or 7 days later. If primed or naïve plants were triggered, the 24 h 4 °C triggering stress was started 5 or 7 days after the end of the priming stimulus 2.5 h after the onset of light. Primed and triggered (PT), only primed (P), only triggered (T) and control plants (C) were harvested at the same time after the 24 h cold stimulus at 4 °C (Fig. 7).

### tAPX_prom_::GUS reporter gene line: construction and analysis

Using the primers CACGTACGGTGGCGAAACG and CACCTCATCAGTTACAAGTGC, a 1468 bp long genomic fragment of Arabidopsis thaliana starting 3 bp upstream of the translation start codon of tAPX (At1g77490), was amplified by PCR and cloned into the GATEWAY vector pENTR D/TOPO (Invitrogen, Carlsbad, U.S.A.) and transferred with LR-Clonase (Invitrogen, Carlsbad, U.S.A.) into the Gateway site of the vector pHGWFS7.0^101^ upstream of the fused cDNAs for GFP and GUS. Following confirmation of the cloning steps by sequencing, *Arabidopsis thaliana var. Col-0* was transformed with the T-DNA using the *Agrobacterium tumefaciens* strain GV3101 (pMP90). Primary transformants were selected on kanamycin and tested fluorometrically for GFP activity^102^. Lines were isolated that segregated for single T-DNA insertions in the T2 generation. GUS histochemistry and quantitative GUS activity analysis were performed with homozygous lines according to standard protocols^103,104^.

### Generation, testing and analysis of estradiol-inducible tAPX and sAPX overexpression and silencing lines

Inducible *tAPX* silencing plants (tAPX-iRNAi) were kindly provided by Shigeru Shigeoka^60^. The inducible lines overexpressing *sAPX* and *tAPX* were generated by amplifying the full length open reading frames (ORF) for *sAPX* and *tAPX* by PCR with gene specific primers (forward: GTTGATCAACAATTAAACACAAAAAC, reverse: ACAAAACCAAGGGTGTGTAGTTATA for *sAPX*; forward: TCAGCTGATAGAAATCATTATCCA, reverse: AAGAAACTCACACTAATCTCAAAATTCT for *tAPX*) from genomic DNA by ligating the PCR products into the pCR8/GW/TOPO vector (Thermo Fisher Scientific, Germany). After control by sequencing, the APX encoding constructs were cloned into the vector pMDC7^105^ downstream of an XVE-inducible promoter based on the GATEWAY cloning technique (LR-reaction; Invitrogen, Carlsbad, U.S.A.). The plasmids, additionally harboring an ORF for the chimeric XVE transcription factor were transferred into *Agrobacterium tumefaciens* GV3101 (pMP90). XVE is activated by estradiol, which leads to the activation of the APX promoter^105^. *Arabidopsis thaliana* Col-0 plants were transformed using the floral dip technique^106^. Transgenic seedlings (T1) were selected on MS agar plates containing 15 µg/ml Hygromycin-B^107^. For confirmation of the T-DNA identity, the lines were tested for the T-DNA insertions by PCR using a forward primer binding to the vector directly upstream of the insert (GGACACGCTGAAGCTAGT) and gene specific reverse primers (same as used for PCR amplification of open reading frames). T2-seedlings were re-tested on Hygromycin B and analyzed for homozygosity by scoring the survival on Hygromycin-B media in the T3-generation.

For activation of the transgene, soil-grown T3 plants were sprayed with 100 µM β-estradiol (dissolved in 1 ml DSMO and diluted 1:125 in H_2_O plus 0.1% (v/v) Tween-20). The transgenic lines for the experiments were selected for strong XVE expression by qPCR with XVE specific primers (Table 1). *tAPX* and *sAPX* transcript levels were recorded with gene specific primers (Table 1) and tAPX and sAPX proteins were detected by Western Blot analysis as described before^24^.

**Table 1:** List of primers.

*Annotation*	*AGI code*	forward	reverse
ACT2	At3g18780	AATCACAGCACTTGCACCAAGC	CCTTGGAGATCCACATCTGCTG
APL3	At4G39210	AAACCGAGAAGTGCCGGATTG	GTTGGATGCTGCATTCTCCCAAG
BAP1	At3G61190	ATCGGATCCCACCAGAGATTACGG	AATCTCGGCCTCCACAAACCAG
COR15A	At2G42540	AACGAGGCCACAAAGAAAGC	CAGCTTCTTTACCCAATGTATCTGC
ORE1	At5G39610	CTTACCATGGAAGGCTAAGATGGG	TTCCAATAACCGGCTTCTGTCG
PAL1	At2G37040	GCAGTGCTACCGAAAGAAGTGG	TGTTCGGGATAGCCGATGTTCC
sAPX	At4g08390	AGAATGGGATTAGATGACAAGGAC	TCCTTCTTTCGTGTACTTCGT
tAPX	At1G77490	GCTAGTGCCACAGCAATAGAGGAG	TGATCAGCTGGTGAAGGAGGTC
YLS8	At5G08290	TTACTGTTTCGGTTGTTCTCCATTT	CACTGAATCATGTTCGAAGCAAGT
ZAT10	At1G27730	TCACAAGGCAAGCCACCGTAAG	TTGTCGCCGACGAGGTTGAATG
XVE	non plant	AGATCACAGACACTTTGATCCACC	GAGAGGATGAGGAGGAGCTGG

For the priming (mimicking) experiments, the plants were grown on soil under the standard growth and priming regimes. Col-0 and the transgenic lines were sprayed with estradiol at the time the priming treatment ended in cold-priming experiments (Fig. 7). To stabilize overexpression and the knock-down effect, the plants were re-treated with estradiol after 3 days.

### Quantitative real-time PCR

RNA extraction, cDNA synthesis, contamination controls, qPCR, standardization and quality control were performed as described before^24^. Each sample was analyzed in triplicates and represents gene expression data from one out of 3–5 independently cultivated biological replicates. The primers used for the qPCR analyses were designed using the QUANTPRIME tool^108^ and are listed in Table 1.

### Statistical analyses

For analysis of variance (ANOVA), Tukey tests (*p* < 0.05) and Student’s t-Tests (*p* < 0.05) were performed using the SPSS23 software package (IBM; New York, U.S.A.) or R (www.r-project.org).

### Primary data

Primary data can be accessed on PrimeDB (https://primedb.mpimp-golm.mpg.de/?sid=reviewer&pid=79721b8c879ec3e00d0a27f966d340fa).

## Supplementary information

Supplements and raw data

## References

[1] Gilmour SJ, Hajela RK, Thomashow MF (1988). Cold-acclimation in *Arabidopsis thaliana*. Plant Physiology.

[2] Guy CL, Niemi KJ, Brambl R (1985). Altered gene-expression during cold-acclimation of spinach. Proceedings of the National Academy of Sciences of the United States of America.

[3] Gray GR, Chauvin LP, Sarhan F, Huner NPA (1997). Cold acclimation and freezing tolerance - A complex interaction of light and temperature. Plant Physiology.

[4] Thomashow MF (1999). Plant cold acclimation: Freezing tolerance genes and regulatory mechanisms. Annual Review of Plant Physiology and Plant Molecular Biology.

[5] Huner NPA (1993). Photosynthesis, photoinhibition and low-temperature acclimation in cold tolerant plants. Photosynthesis Research.

[6] Hurry V, Strand A, Furbank R, Stitt M (2000). The role of inorganic phosphate in the development of freezing tolerance and the acclimatization of photosynthesis to low temperature is revealed by the *pho* mutants of *Arabidopsis thaliana*. Plant Journal.

[7] Huner NPA (2013). Shedding some light on cold acclimation, cold adaptation, and phenotypic plasticity. Botany-Botanique.

[8] Ensminger I, Busch F, Huner NPA (2006). Photostasis and cold acclimation: sensing low temperature through photosynthesis. Physiologia Plantarum.

[9] Steponkus PL (1984). Role of the plasma membrane in freezing injury and cold acclimation. Annual Review of Plant Physiology.

[10] Guo X, Xu S, Chong K (2017). Cold signal shuttles from membrane to nucleus. Molecular Cell.

[11] Kaur N, Gupta AK (2005). Signal transduction pathways under abiotic stresses in plants. Current Science.

[12] BMEL. Ernte 2017 - Menge und Preise. *Bundesministerium für Ernährung und Landwirtschaft* (2017).

[13] Degenkolbe T (2012). Differential remodeling of the lipidome during cold acclimation in natural accessions of *Arabidopsis thaliana*. Plant Journal.

[14] Klotke J, Kopka J, Gatzke N, Heyer AG (2004). Impact of soluble sugar concentrations on the acquisition of freezing tolerance in accessions of *Arabidopsis thaliana* with contrasting cold adaptation - evidence for a role of raffinose in cold acclimation. Plant Cell and Environment.

[15] Vega SE, del Rio AH, Bamberg JB, Palta JP (2004). Evidence for the up-regulation of stearoyl-ACP (A9) desaturase gene expression during cold acclimation. American Journal of Potato Research.

[16] Strand Å (1999). Acclimation of Arabidopsis leaves developing at low temperature. Increasing cytoplasmic volume accompanies increased activities of enzymes in the Calvin Cycle and in the sucrose-biosynthesis pathway. Plant Physiology.

[17] Arae T (2017). Co-ordinated regulations of mRNA synthesis and decay during cold acclimation in Arabidopsis cells. Plant and Cell Physiology.

[18] Caldana C (2011). High-density kinetic analysis of the metabolomic and transcriptomic response of Arabidopsis to eight environmental conditions. Plant Journal.

[19] Thomashow MF, Gilmour SJ, Stockinger EJ, Jaglo-Ottosen KR, Zarka DG (2001). Role of the Arabidopsis CBF transcriptional activators in cold acclimation. Physiologia Plantarum.

[20] Jackson MW, Stinchcombe JR, Korves TM, Schmitt J (2004). Costs and benefits of cold tolerance in transgenic *Arabidopsis thaliana*. Molecular Ecology.

[21] Zuther E, Juszczak I, Lee YP, Baier M, Hincha DK (2015). Time-dependent deacclimation after cold acclimation in *Arabidopsis thaliana* accessions. Scientific Reports.

[22] Kalberer SR, Wisniewski M, Arora R (2006). Deacclimation and reacclimation of cold-hardy plants: Current understanding and emerging concepts. Plant Science.

[23] Baier, M., Bittner, A., Prescher, A. & van Buer, J. Preparing plants for improved cold tolerance by priming. *Plant Cell and Environment* (2018, online first). 10.1111/pce.13394 29974962

[24] van Buer, J., Cvetkovic, J. & Baier, M. Cold regulation of plastid ascorbate peroxidases serves as a priming hub controlling ROS signaling in *Arabidopsis thaliana*. *Bmc Plant Biology* **16** (2016). 10.1186/s12870-016-0856-7 PMC4955218 27439459

[25] Hilker, M. *et al*. Priming and memory of stress responses in organisms lacking a nervous system. *Biological Reviews of the Cambridge Philosophical Society* **91** (2016). 10.1111/brv.12215 26289992

[26] Hahn A (2013). Plant core environmental stress response genes are systemically coordinated during abiotic stresses. International Journal Molecular Sciences.

[27] Op Den Camp RGL (2003). Rapid induction of distinct stress responses after the release of singlet oxygen in arabidopsis. Plant Cell.

[28] Laloi C (2007). Cross-talk between singlet oxygen- and hydrogen peroxide-dependent signaling of stress responses in *Arabidopsis thaliana*. Proceedings of the National Academy of Sciences of the United States of America.

[29] Estavillo GM (2011). Evidence for a SAL1-PAP chloroplast retrograde pathway that functions in drought and high light signaling in Arabidopsis. Plant Cell.

[30] Lee KP, Kim C, Landgraf F, Apel K (2007). EXECUTER1- and EXECUTER2-dependent transfer of stress-related signals from the plastid to the nucleus of *Arabidopsis thaliana*. Proceedings of the National Academy of Sciences of the United States of America.

[31] Conrath U, Beckers GJM, Langenbach CJG, Jaskiewicz MR (2015). Priming for enhanced defense. Annual Review of Phytopathology.

[32] Hossain MA (2017). Heat or cold priming-induced cross-tolerance to abiotic stresses in plants: key regulators and possible mechanisms. Protoplasma.

[33] Thellier M, Luttge U (2013). Plant memory: a tentative model. Plant Biology.

[34] Lozano-Duran R, Zipfel C (2015). Trade-off between growth and immunity: role of brassinosteroids. Trends in Plant Science.

[35] van Hulten M, Pelser M, van Loon LC, Pieterse CMJ, Ton J (2006). Costs and benefits of priming for defense in Arabidopsis. Proceedings of the National Academy of Sciences of the United States of America.

[36] Crisp PA, Ganguly D, Eichten SR, Borevitz JO, Pogson BJ (2016). Reconsidering plant memory: Intersections between stress recovery, RNA turnover, and epigenetics. Science Advances.

[37] Boyes DC (2001). Growth stage-based phenotypic analysis of Arabidopsis: a model for high throughput functional genomics in plants. Plant Cell.

[38] Pena-Ahumada A, Kahmann U, Dietz KJ, Baier M (2006). Regulation of peroxiredoxin expression versus expression of Halliwell-Asada-Cycle enzymes during early seedling development of Arabidopsis thaliana. Photosynthesis Research.

[39] Heiber I, Cai W, Baier M (2014). Linking chloroplast antioxidant defense to carbohydrate availability: the transcript abundance of stromal ascorbate peroxidase is sugar-controlled via ascorbate biosynthesis. Molecular Plant.

[40] Eastmond PJ (2000). Postgerminative growth and lipid catabolism in oilseeds lacking the glyoxylate cycle. Proceedings of the National Academy of Sciences of the United States of America.

[41] Wang Y, Hua J (2009). A moderate decrease in temperature induces *COR15a* expression through the CBF signaling cascade and enhances freezing tolerance. Plant Journal.

[42] Lee BH, Henderson DA, Zhu JK (2005). The Arabidopsis cold-responsive transcriptome and its regulation by ICE1. Plant Cell.

[43] Steponkus PL, Uemura M, Joseph RA, Gilmour SJ, Thomashow MF (1998). Mode of action of the *COR15a* gene on the freezing tolerance of *Arabidopsis thaliana*. Proceedings of the National Academy of Sciences of the United States of America.

[44] Laloi C, Przybyla D, Apel K (2006). A genetic approach towards elucidating the biological activity of different reactive oxygen species in *Arabidopsis thaliana*. Journal of Experimental Botany.

[45] Wan F (2014). Heterologous expression of Arabidopsis C-repeat binding factor 3 (AtCBF3) and cold-regulated 15A (AtCOR15A) enhanced chilling tolerance in transgenic eggplant (*Solanum melongena* L.). Plant Cell Reports.

[46] Baker SS, Wilhelm KS, Thomashow MF (1994). The 5′-region of *Arabidopsis thaliana Cor15a* has *cis*-acting elements that confer cold-regulated, drought-regulated and ABA-regulated gene expression. Plant Molecular Biology.

[47] Villand P, Olsen OA, Kleczkowski LA (1993). Molecular characterization of multiple cDNA clones for ADP-glucose pyrophosphorylase from *Arabidopsis thaliana*. Plant Molecular Biology.

[48] Sokolov LN, Dejardin A, Kleczkowski LA (1998). Sugars and light/dark exposure trigger differential regulation of ADP-glucose pyrophosphorylase genes in *Arabidopsis thaliana* (thale cress). Biochemical Journal.

[49] Rook F (2001). Impaired sucrose-induction mutants reveal the modulation of sugar-induced starch biosynthetic gene expression by abscisic acid signalling. Plant Journal.

[50] Balazadeh S (2010). A gene regulatory network controlled by the NAC transcription factor ANAC092/AtNAC2/ORE1 during salt-promoted senescence. Plant Journal.

[51] Kim JH, Chung KM, Woo HR (2011). Three positive regulators of leaf senescence in Arabidopsis, ORE1, ORE3 and ORE9, play roles in crosstalk among multiple hormone-mediated senescence pathways. Genes & Genomics.

[52] Hanaoka H (2002). Leaf senescence and starvation-induced chlorosis are accelerated by the disruption of an Arabidopsis autophagy gene. Plant Physiology.

[53] Woo HR, Kim JH, Nam HG, Lim PO (2004). The delayed leaf senescence mutants of Arabidopsis, *ore*1, *ore*3, and *ore*9 are tolerant to oxidative stress. Plant and Cell Physiology.

[54] Qiu, K. *et al*. EIN3 and ORE1 accelerate degreening during ethylene-mediated leaf senescence by directly activating chlorophyll catabolic genes in Arabidopsis. *Plos Genetics* **11** (2015). 10.1371/journal.pgen.1005399 PMC4517869 26218222

[55] Kim JH (2009). Trifurcate feed-forward regulation of age-dependent cell death involving miR164 in Arabidopsis. Science.

[56] Pitsch NT, Witsch B, Baier M (2010). Comparison of the chloroplast peroxidase system in the chlorophyte *Chlamydomonas reinhardtii*, the bryophyte *Physcomitrella patens*, the lycophyte *Selaginella moellendorffii* and the seed plant *Arabidopsis thaliana*. BMC Plant Biology.

[57] Maruta T, Sawa Y, Shigeoka S, Ishikawa T (2016). Diversity and evolution of ascorbate peroxidase functions in chloroplasts: More than just a classical antioxidant enzyme?. Plant Cell Physiology.

[58] Danna CH (2003). Thylakoid-bound ascorbate peroxidase mutant exhibits impaired electron transport and photosynthetic activity. Plant Physiology.

[59] Kangasjärvi S (2008). Diverse roles for chloroplast stromal and thylakoid-bound ascorbate peroxidases in plant stress responses. Biochemical Journal.

[60] Maruta T (2012). H_2_O_2_-triggered retrograde signaling from chloroplasts to nucleus plays specific role in response to stress. Journal of Biological Chemistry.

[61] Maruta T (2010). Arabidopsis chloroplastic ascorbate peroxidase isoenzymes play a dual role in photoprotection and gene regulation under photooxidative stress. Plant and Cell Physiology.

[62] Zuo JR, Niu QW, Chua NH (2000). An estrogen receptor-based transactivator XVE mediates highly inducible gene expression in transgenic plants. Plant Journal.

[63] Hossain MA, Asada K (1984). Inactivation of ascorbate peroxidase in spinach chloroplasts on dark addition of hydrogen peroxide: Its protection by ascorbate. Plant Cell and Environment.

[64] Kitajima S (2008). Hydrogen peroxide-mediated inactivation of two chloroplastic peroxidases, ascorbate peroxidase and 2-Cys peroxiredoxin. Photochemistry and Photobiology.

[65] Baier, M., Pitsch, N. T., Mellenthin, M. & Guo, W. Regulation of genes encoding chloroplast antioxidant enzymes in comparison to regulation of the extra-plastidic antioxidant defense system. in *Ascorbate-glutathione pathway and stress tolerance in plants* (eds N. A. Anjum, M. -T. Chan, & S. Umar) pp 337–386 (2010).

[66] Dekker JP, Boekema EJ (2005). Supramolecular organization of thylakoid membrane proteins in green plants. Biochimica Et Biophysica Acta-Bioenergetics.

[67] Mano J, Hideg E, Asada K (2004). Ascorbate in thylakoid lumen functions as an alternative electron donor to photosystem II and photosystem I. Archives Biochemistry Biophysics.

[68] Polle A (1996). Mehler reaction: Friend or foe in photosynthesis?. Botanica Acta.

[69] Mehler AH (1951). Studies on reactions of illuminated chloroplasts .1. Mechanism of the reduction of oxygen and other Hill reagents. Archives of Biochemistry and Biophysics.

[70] Mano J, Ohno C, Domae Y, Asada K (2001). Chloroplastic ascorbate peroxidase is the primary target of methylviologen-induced photooxidative stress in spinach leaves: its relevance to monodehydroascorbate radical detected with *in vivo* ESR. Biochimica et Biophysica Acta-Bioenergetics.

[71] Miyake C, Asada K (1996). Inactivation mechanism of ascorbate peroxidase at low concentrations of ascorbate: Hydrogen peroxide decomposes compound I of ascorbate peroxidase. Plant Cell Physiology.

[72] Kitajima S, Shimaoka T, Kurioka M, Yokota A (2007). Irreversible cross-linking of heme to the distal tryptophan of stromal ascorbate peroxidase in response to rapid inactivation by H_2_O_2_. FEBS Journal.

[73] Exposito-Rodriguez M, Laissue PP, Yvon-Durocher G, Smirnoff N, Mullineaux PM (2017). Photosynthesis-dependent H_2_O_2_ transfer from chloroplasts to nuclei provides a high-light signalling mechanism. Nature Communications.

[74] Rossel JB (2007). Systemic and intracellular responses to photooxidative stress in Arabidopsis. Plant Cell.

[75] Zhu Y, Yang H, Mang HG, Hua J (2011). Induction of *BAP1* by a moderate decrease in temperature is mediated by ICE1 in Arabidopsis. Plant Physiology.

[76] Mittler R (2006). Gain- and loss-of-function mutations in *Zat10* enhance the tolerance of plants to ablotic stress. FEBS Letters.

[77] Hua J, Grisafi P, Cheng SH, Fink GR (2001). Plant growth homeostasis is controlled by the Arabidopsis *BON1* and *BAP1* genes. Genes & Development.

[78] Baxter A, Mittler R, Suzuki N (2014). ROS as key players in plant stress signalling. Journal of Experimental Botany.

[79] Foyer CH, Noctor G (2016). Stress-triggered redox signalling: what’s in pROSpect?. Plant Cell and Environment.

[80] Springthorpe V, Penfield S (2015). Flowering time and seed dormancy control use external coincidence to generate life history strategy. eLife.

[81] Penfield S, Springthorpe V (2012). Understanding chilling responses in Arabidopsis seeds and their contribution to life history. Philosophical Transactions of the Royal Society B-Biological Sciences.

[82] Olivas NHD (2017). Natural variation in life history strategy of *Arabidopsis thaliana* determines stress responses to drought and insects of different feeding guilds. Molecular Ecology.

[83] Sung SB, Amasino RM (2004). Vernalization and epigenetics: how plants remember winter. Current Opinion in Plant Biology.

[84] Bastow R (2004). Vernalization requires epigenetic silencing of FLC by histone methylation. Nature.

[85] Onouchi H, Coupland G (1998). The regulation of flowering time of Arabidopsis in response to daylength. Journal of Plant Research.

[86] Lievre M, Granier C, Guedon Y (2016). Identifying developmental phases in the *Arabidopsis thaliana* rosette using integrative segmentation models. New Phytologist.

[87] Alcazar R, Reymond M, Schmitz G, de Meaux J (2011). Genetic and evolutionary perspectives on the interplay between plant immunity and development. Current Opinion in Plant Biology.

[88] Baier M, Dietz K-J (1996). Primary structure and expression of plant homologues of animal and fungal thioredoxin-dependent peroxide reductases and bacterial alkyl hydroperoxide reductases. Plant Molecular Biology.

[89] Bartelheimer, M., Schmid, C., Storf, J., Hell, K. & Bauer, S. Interspecific competition in *Arabidopsis thaliana*: A knowledge gap is starting to close. *Progress in Botany*, 303–319 (2015).

[90] Barenfaller K (2016). A long photoperiod relaxes energy management in Arabidopsis leaf six. Current Plant Biology.

[91] Rudnik R, Bulcha JT, Reifschneider E, Ellersiek U, Baier M (2017). Specificity versus redundancy in the RAP2.4 transcription factor family of *Arabidopsis thaliana*: transcriptional regulation of genes for chloroplast peroxidases. Bmc Plant Biology.

[92] Hiltscher H (2014). The radical induced cell death protein 1 (RCD1) supports transcriptional activation of genes for chloroplast antioxidant enzymes. Frontiers in Plant Science.

[93] Heiber I (2007). The redox imbalanced mutants of Arabidopsis differentiate signaling pathways for redox regulation of chloroplast antioxidant enzymes. Plant Physiology.

[94] Asada K (2000). The water-water cycle as alternative photon and electron sinks. Philosophical Transactions of the Royal Society London B-Series Biological Sciences.

[95] Noctor G, Reichheld JP, Foyer CH (2018). ROS-related redox regulation and signaling in plants. Seminars in Cell & Developmental Biology.

[96] Asada K (1999). The water-water cycle in chloroplasts: Scavenging of active oxygen and dissipation of excess photons. Annual Review Plant Physiology Plant Molecular Biology.

[97] Duan M (2012). Antisense-mediated suppression of tomato thylakoidal ascorbate peroxidase influences anti-oxidant network during chilling stress. Plant Physiology Biochemistry.

[98] Sun WH (2010). Overexpression of tomato *tAPX* gene in tobacco improves tolerance to high or low temperature stress. Biologia Plantarum.

[99] Murgia I (2004). *Arabidopsis thaliana* plants overexpressing thylakoidal ascorbate peroxidase show increased resistance to Paraquat-induced photooxidative stress and to nitric oxide-induced cell death. Plant Journal.

[100] Juszczak I, Rudnik R, Pietzenuk B, Baier M (2012). Natural genetic variation in the expression regulation of the chloroplast antioxidant system among *Arabidopsis thaliana* accessions. Physiologia Plantarum.

[101] Karimi M, Inze D, Depicker A (2002). GATEWAY^TM^ vectors for Agrobacterium-mediated plant transformation. Trends in Plant Science.

[102] Mellenthin M, Ellersiek U, Börger A, Baier M (2014). Expression of the Arabidopsis sigma factor SIG5 is photoreceptor and photosynthesis controlled. Plants.

[103] Abel, S. & Theologis, A. In *Arabidopsis Protocols: Methods in Molecular Biology* Vol. 82 (eds Martinez-Zapater J. M. & Salinas J.) (Humana Press, 1998).

[104] Jefferson RA, Kavanagh TA, Bevan MW (1987). GUS fusion: β-glucuronidase as a sensitive and versatile gene fusion marker in higher plants. EMBO.

[105] Curtis MD, Grossniklaus U (2003). A gateway cloning vector set for high-throughput functional analysis of genes in planta. Plant Physiology.

[106] Bernhardt K, Vigelius SK, Wiese J, Linka N, Weber APM (2012). Agrobacterium-mediated *Arabidopsis thaliana* transformation: an overview of T-DNA binary vectors, floral dip and screening for homzygeous lines. Journal of Endocytobiosis and Cell Research.

[107] Harrison SJ (2006). A rapid and robust method of identifying transformed *Arabidopsis thaliana* seedlings following floral dip transformation. Plant Methods.

[108] Arvidsson S, Kwasniewski M, Riano-Pachon DM, Mueller-Roeber B (2008). QuantPrime - a flexible tool for reliable high-throughput primer design for quantitative PCR. BMC Bioinformatics.

