# Supplementary material for: Cold-priming of chloroplast ROS signalling is developmentally regulated and is locally controlled at the thylakoid membrane
Source: Sci Rep. 2019 Feb 28;9:3022. doi: 10.1038/s41598-019-39838-3 (PMC6395587; doi:10.1038/s41598-019-39838-3)

Supplements

Cold-priming of chloroplast ROS signalling is developmentally regulated and is locally controlled at the thylakoid membrane

Jörn van Buer, Andreas Prescher, Margarete Baier

Supplement 1

Cold-priming of chloroplast ROS signalling is developmentally regulated and is locally controlled at the thylakoid membrane

Jörn van Buer, Andreas Prescher, Margarete Baier

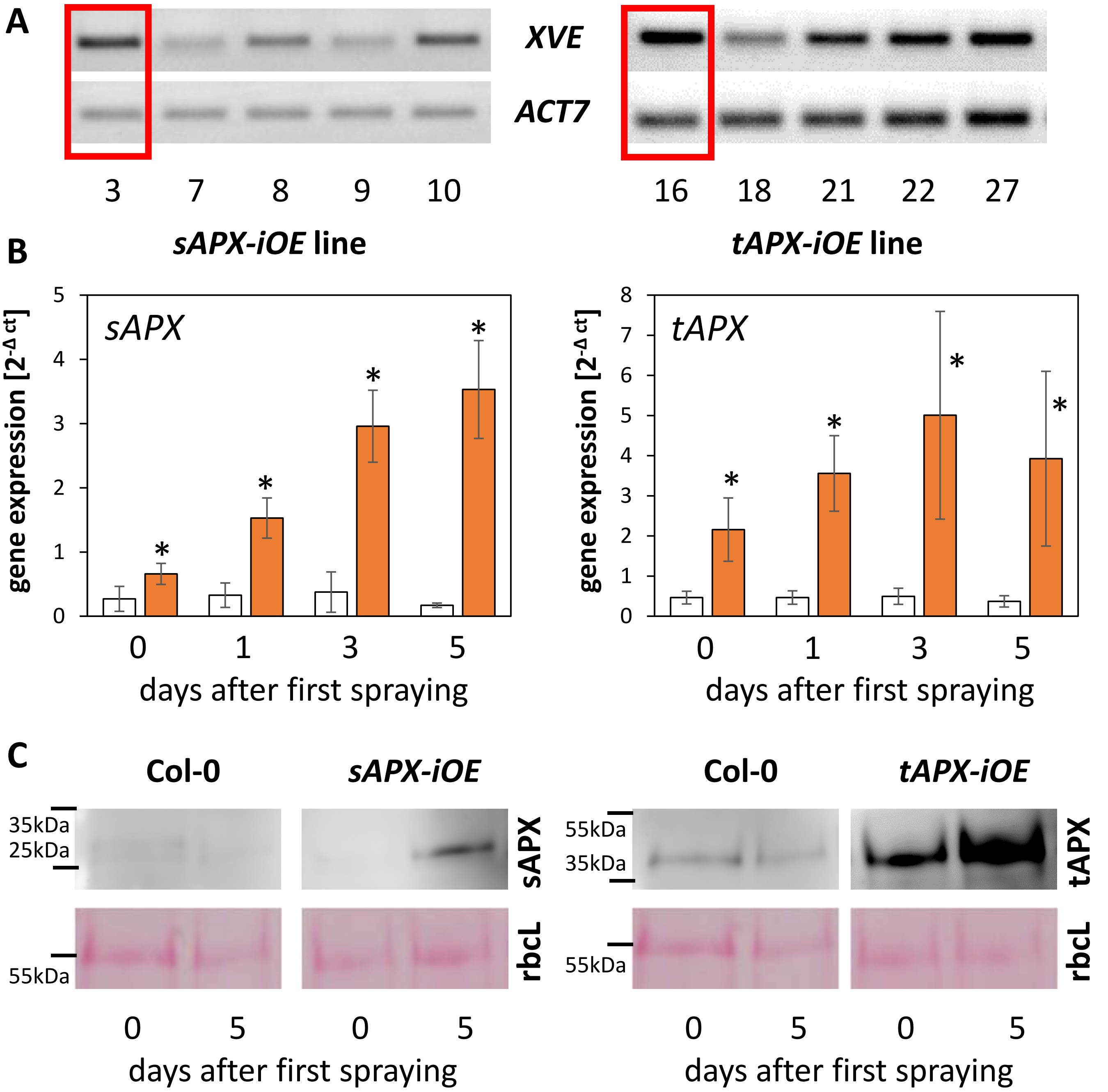

Supplement 1: Selection and pre-testing of the *sAPX-iOE* and *tAPX-iOE* lines. (A) Screening of independent, homozygous transgenic lines for the expression intensity of the transactivator *XVE*. The lines used for subsequent experiments are boxed red. **(B)** Transcript levels of *sAPX* and *tAPX* in the over-expression lines after spraying with estradiol at day 0 and 3 (orange) relative to the transcript levels in parallel grown Col-0 plants (white). Asterisks label transcript levels significantly higher than the levels in Col-0 plants (t-Test $p<0.05$; $n=4$). **(C)** sAPX and tAPX protein amounts in the same iOE-lines 0 and 5 days after the first spraying. Gel loading was controlled by Ponceau S staining of the Western blot membrane. The strongest band, representing the large subunit of RUBISCO, rbcL, is shown.

Supplement 2

Cold-priming of chloroplast ROS signalling is developmentally regulated and is locally controlled at the thylakoid membrane

Jörn van Buer, Andreas Prescher, Margarete Baier

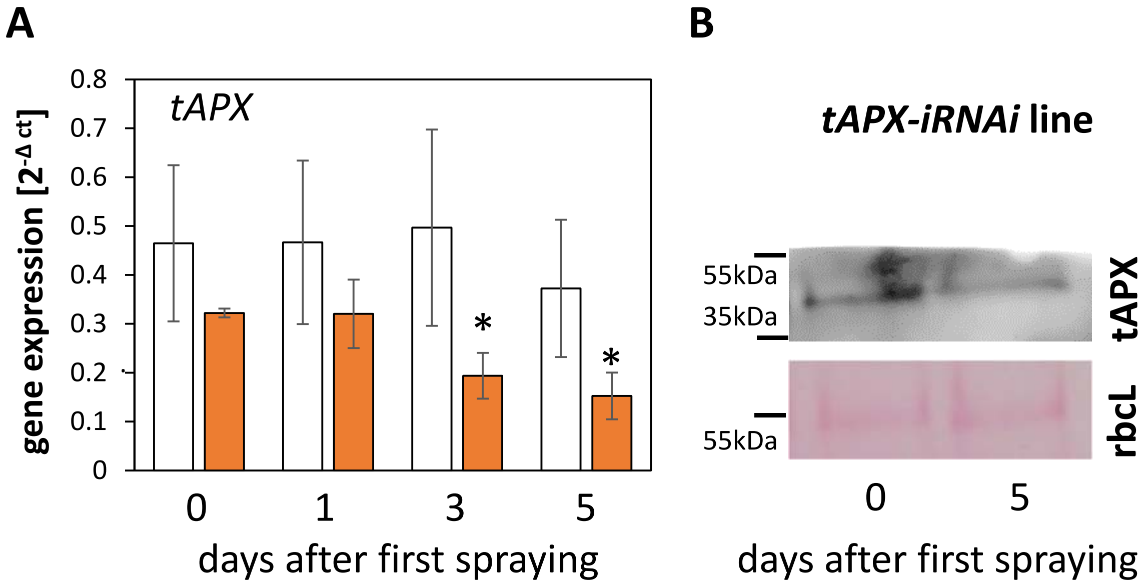

Supplement 2: Pre-testing of the *tAPX-iRNAi* line. (A) Transcript levels of *tAPX* in the inducible RNAi after spraying with estradiol at day 0 and 3 (orange) relative to the transcript levels in parallel grown Col-0 plants (white). Stars label transcript levels significantly higher than the levels in Col-0 plants (t-Test $p<0.05$; n=4). **(B)** tAPX protein amounts in the same RNAi-line 0 and 5 days after the first spraying. Gel loading was controlled by Ponceau S staining of the Western blot membrane. The strongest band, representing the large subunit of RUBISCO, rbcL, is shown.

Raw images – Western Blot A **van Buer et al.**

Cold-priming of chloroplast ROS signalling is developmentally regulated and is locally controlled at the thylakoid membrane

Jörn van Buer, Andreas Prescher, Margarete Baier

All Western Blot data and Ponceau S-staining data were taken from the same membrane, which was first stained with Ponceau-S and than treated with antibodies and the detection agent.

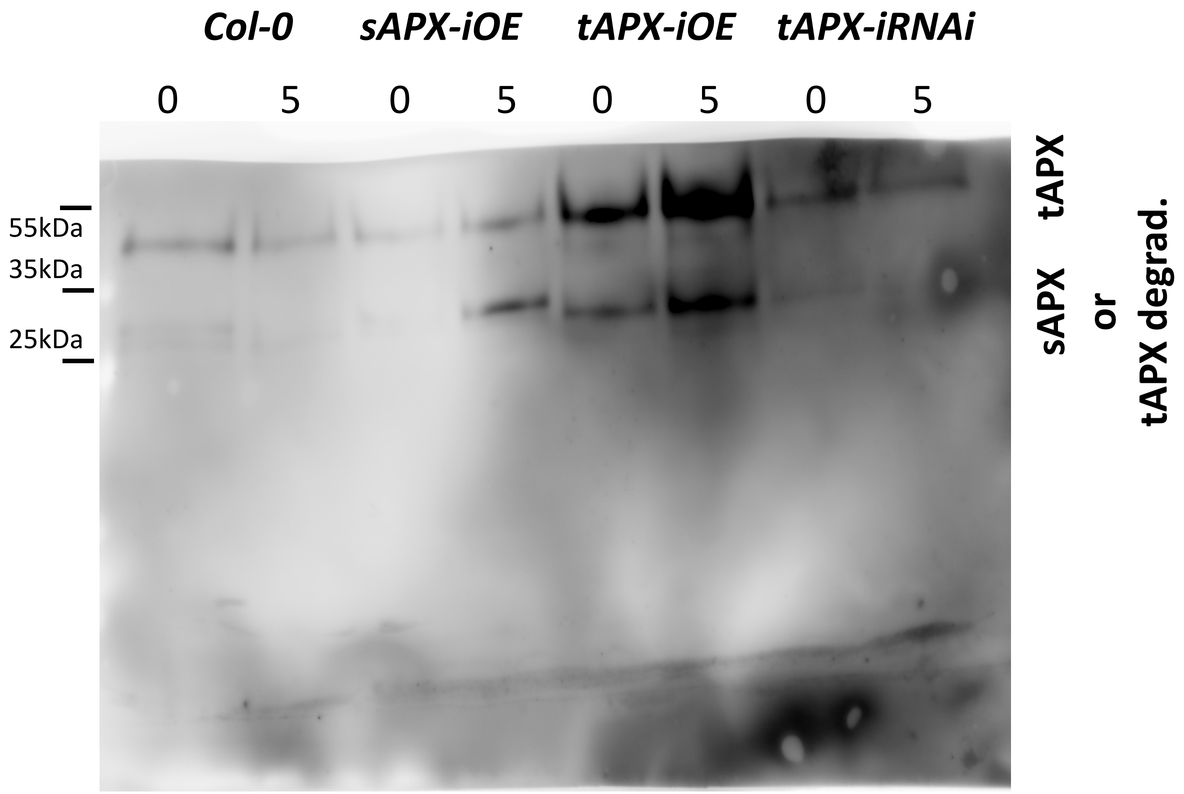

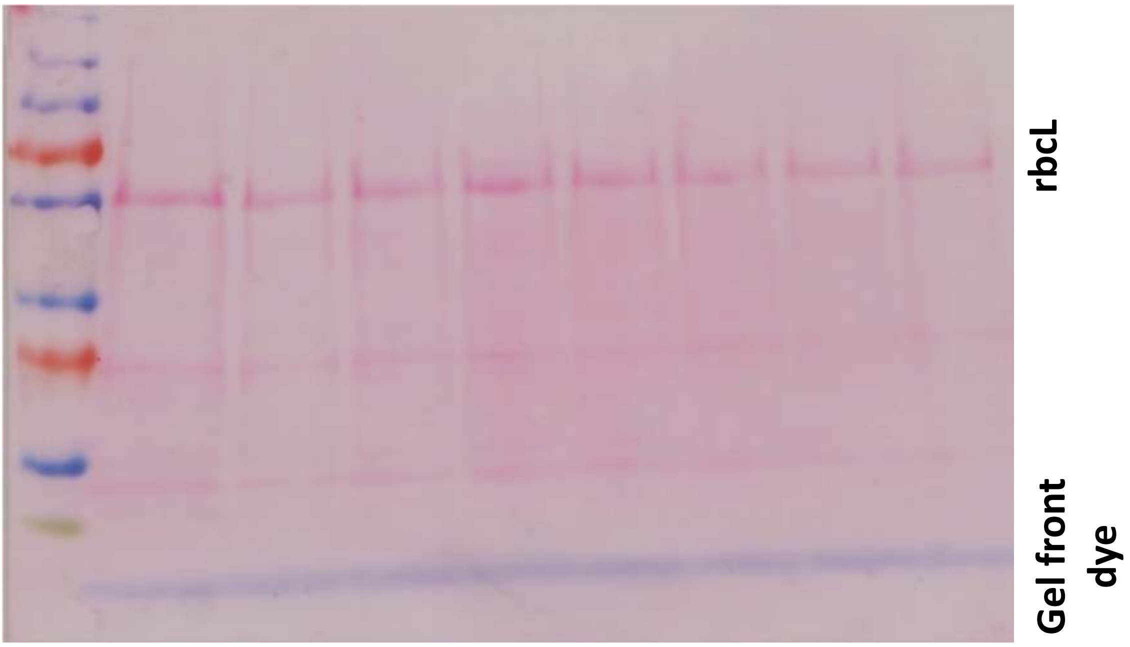

Cold-priming of chloroplast ROS signalling is developmentally regulated and is locally controlled at the thylakoid membrane

Jörn van Buer, Andreas Prescher, Margarete Baier

The Western-Blot parts shown in Suppl. 1 and Suppl. 2 are boxed in red.

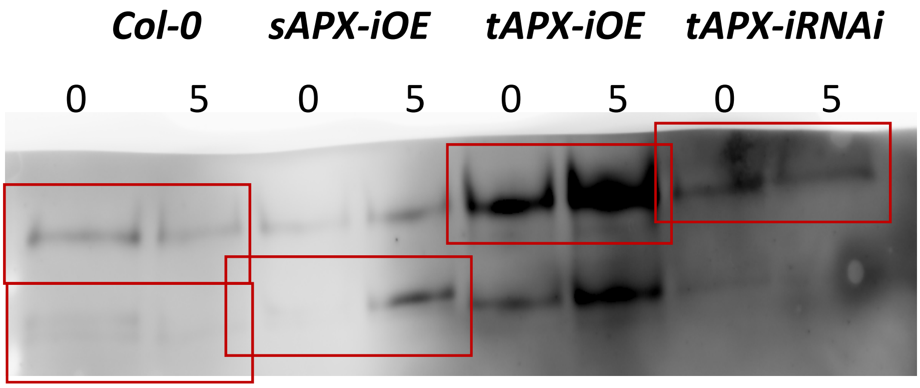

The lower band in the sAPX-iOE line is smaller than sAPX and has been interpreted as tAPX degradation product / artefact, which accumulates proportional with tAPX.

Cold-priming of chloroplast ROS signalling is developmentally regulated and is locally controlled at the thylakoid membrane

Jörn van Buer, Andreas Prescher, Margarete Baier

From populations of independent transgenic lines, the information on the first 5 lines is shown in supplement 2. The ACT7- and XVE-PCRs were run for each iOE construct on the same gel in the same order.

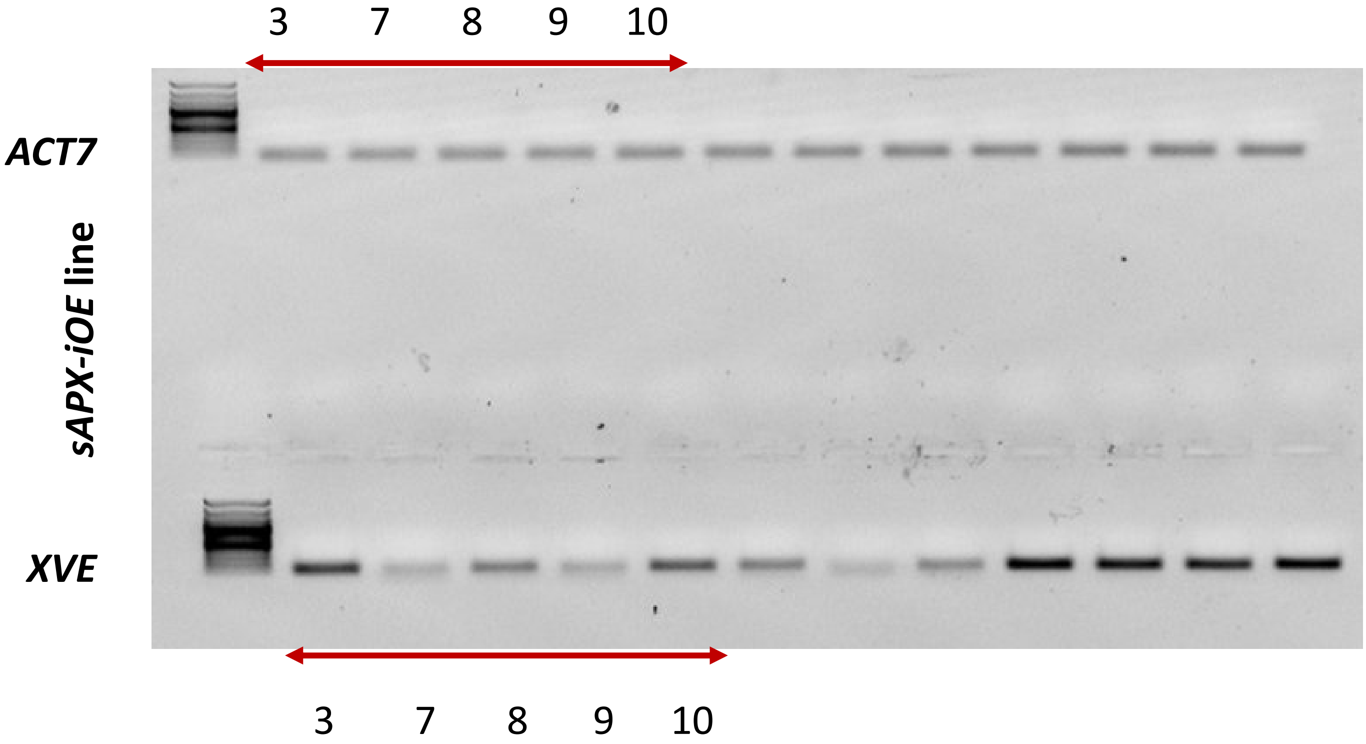

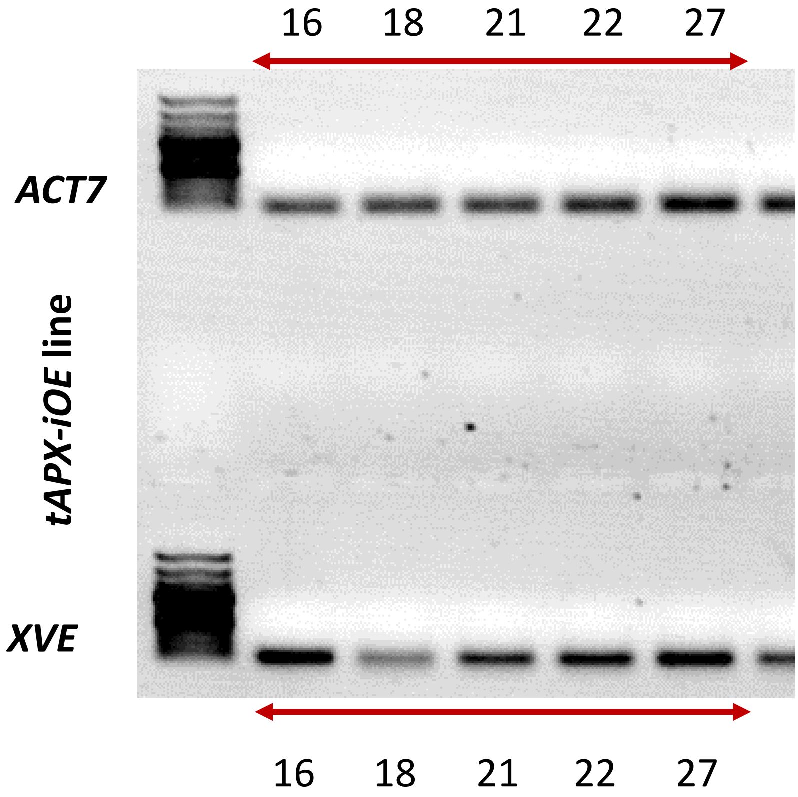

Supplement: Supplementary file 1 — Supplements and raw data [file 41598_2019_39838_MOESM1_ESM.pdf]
